# Left ventricular active strain energy density is a promising new measure of systolic function

**DOI:** 10.1038/s41598-022-15509-8

**Published:** 2022-07-26

**Authors:** David H. MacIver, Peter Agger, Jonathan C. L. Rodrigues, Henggui Zhang

**Affiliations:** 1 Department of Cardiology, Taunton & Somerset Hospital, Musgrove Park, UK; 2 Biological Physics Group, Department of Astronomy and Physics, University of Manchester, Manchester, UK; 3 Comparative Medicine Lab, Department of Clinical Medicine, Aarhus University, Aarhus, Denmark; 4 Department of Radiology, Royal United Hospital Bath NHS Trust, Bath, UK; 5 Department of Health, University of Bath, Bath, UK

**Keywords:** Biological techniques, Biophysics, Structural biology, Cardiology, Medical research, Engineering, Physics, Physiology, Cardiovascular biology, Circulation

## Abstract

The left ventricular ejection fraction does not accurately predict exercise capacity or symptom severity and has a limited role in predicting prognosis in heart failure. A better method of assessing ventricular performance is needed to aid understanding of the pathophysiological mechanisms and guide management in conditions such as heart failure. In this study, we propose two novel measures to quantify myocardial performance, the global longitudinal active strain energy (GLASE) and its density (GLASED) and compare them to existing measures in normal and diseased left ventricles. GLASED calculates the work done per unit volume of muscle (energy density) by combining information from myocardial strain and wall stress (contractile force per unit cross sectional area). Magnetic resonance images were obtained from 183 individuals forming four cohorts (normal, hypertension, dilated cardiomyopathy, and cardiac amyloidosis). GLASE and GLASED were compared with the standard ejection fraction, the corrected ejection fraction, myocardial strains, stroke work and myocardial forces. Myocardial shortening was decreased in all disease cohorts. Longitudinal stress was normal in hypertension, increased in dilated cardiomyopathy and severely decreased in amyloid heart disease. GLASE was increased in hypertension. GLASED was mildly reduced in hypertension (1.39 ± 0.65 kJ/m^3^), moderately reduced in dilated cardiomyopathy (0.86 ± 0.45 kJ/m^3^) and severely reduced in amyloid heart disease (0.42 ± 0.28 kJ/m^3^) compared to the control cohort (1.94 ± 0.49 kJ/m^3^). GLASED progressively decreased in the hypertension, dilated cardiomyopathy and cardiac amyloid cohorts indicating that mechanical work done and systolic performance is severely reduced in cardiac amyloid despite the relatively preserved ejection fraction. GLASED provides a new technique for assessing left ventricular myocardial health and contractile function.

## Introduction

Left ventricular (LV) ejection fraction (EF) does not accurately predict symptom severity^1^ or exercise capacity^2^ in heart failure and may not reflect ventricular performance accurately^3,4^. EF predicted prognosis following myocardial infarction in the thrombolysis era^5,6^ but is unreliable in modern times^7–9^.

The EF may be inaccurate because it relies on data obtained from the lumen alone and in so doing loses valuable information relating to myocardial structure and mechanics. In silico-modelling studies^10–15^ show that midwall circumferential myocardial shortening, longitudinal shortening, myocardial mural thickness, ventricular internal diameter, and ventricular length each influence the EF independently (Table 1). Clinical studies confirm the importance of these confounding effects of geometric changes on EF and explain its shortcomings in assessing ventricular performance^15–18^.

**Table 1:** Effect of an increase in the input variables on ejection fraction, corrected ejection fraction, pressure-strain loop and ASED (CASED/GLASED).

Variable	EF	EF_c_	PSL	CASED	GLASED
↑LS	↑↑	↑↑	↑↑	–	↑↑↑
↑MCS	↑↑↑	↑↑↑	–	↑↑↑	–
↑EDWT	↑↑↑	–	–	↓↓	↓↓
↑LVIDd	↓↓	–	–	↑↑	↑↑
↑LV length	↓	–	–	–	–
↑Pressure	–	–	↑↑	↑↑	↑↑

Increase of longitudinal shortening (LS), midwall circumferential shortening (MCS), end-diastolic wall thickness (EDWT), left ventricular internal dimensions in diastole (LVIDd), left ventricular (LV) length and ventricular pressure generation have differing effects on EF^10,12,14^, EF_c_^17,19^, PSL (pressure-strain loop area), GLASED and midwall CASED. MCS has a greater effect on EF, EF_c_ and stroke volume compared with longitudinal shortening^11^.

Note the opposing impacts of EDWT and LVIDd on EF and GLASED and the absence of an effect of EDWT, LVIDd and LV length on EF_c_. There is an apparent lack of a *direct* pressure effect on EF and EF_c_. despite the effect of hemodynamic load on strain. The effect of changes in pressure generation on EF are masked through remodelling with changes in EDWT and LVIDd.

The limitations of EF described above have resulted in a search for improved measures of ventricular performance. For example, the influence of confounding structural changes on the EF can be removed by calculating the corrected ejection fraction (EF_c_)^17,19^.

Myocardial strain is an improvement on EF at measuring actual myocardial function^20^ A reduced myocardial strain occurs in most heart muscle disorders including heart failure with a preserved EF (HFpEF), hypertensive heart disease, aortic stenosis, hypertrophic cardiomyopathy and concentric hypertrophy despite the usually preserved EF^14,21^ However, the major drawback of strain analysis is that it fails consider haemodynamic load and, therefore, would not be expected to accurately measure myocardial function^9,20^.

Haemodynamic load can be assessed by calculating contractile stress. Contractile stress measures the active force generated per unit cross-sectional area and results in cardiomyocyte contraction with associated longitudinal and circumferential shortening, radial thickening, and intraventricular pressure generation. Contractile stress incorporates information from LV mural thickness, cavity size and, crucially, pressure generation but does not provide information on the resultant deformation.

Combining the information from stress and strain should provide a superior measure of systolic function. The elastic strain energy density is the energy per unit volume stored in a material under external loading and is calculated by integrating data from both the stress and strain. We can define myocardial active strain energy (ASED) as the mechanical energy (work done) per unit volume of muscle necessary to deform the myocardium and generate systolic pressure.

Global longitudinal active strain energy density (GLASED) and midwall circumferential strain energy density (CASED) provide an estimate of the mechanical work done per unit volume of myocardium in association with the stress and strain in the longitudinal and circumferential directions, respectively. Active strain energy (ASE) estimates the energy produced by the whole LV myocardium. Global longitudinal active strain energy (GLASE) and midwall circumferential strain energy (CASE) estimates the energy produced in the longitudinal and circumferential directions, respectively. The measurement of ASE and ASED would be expected to be excellent measures of ventricular and myocardial health and performance because they combine data from contractile stress (force generated per unit cross-sectional area) and percent shortening while also supplying an estimation of the energy produced by the myocardium during systole.

The EF has been described as ‘a measure of desperation’^3^ and a recent editorial declared that it is ‘time to leave diagnostics based on ejection fraction’^4^ because of the significant limitations described above. The search for better measures of LV performance is extremely important, as such knowledge would have a major impact on understanding conditions such as heart failure, modify treatment recommendations and provide valuable prognostic information.

Measuring ASED avoids the weaknesses of both EF and strain by accounting for both the confounding effect of abnormal geometric patterns (mural thickness and ventricular cavity size) and the pressure generation (loading conditions).

Our primary hypothesis was that ASED (i.e. GLASED and CASED) would be significantly different between the disease cohorts and be more closely aligned to previously published mortality data compared to current methods. Therefore, we assessed ASED in individuals with DCM, hypertension, and amyloid heart disease, and contrasted them with healthy controls, as each of these cohorts have distinct geometric differences and haemodynamic loading conditions. We employed cardiac magnetic resonance (CMR) for the study because it is the gold standard for assessing left ventricular structure.

## Clinical perspective

Current measures of left ventricular performance such as ejection fraction and myocardial strain have important shortcomings. These limitations are attributable to the confounding effects of abnormal geometric changes and the loading conditions. This study introduces a novel measure of left ventricular systolic function called the global longitudinal active strain energy density (GLASED). GLASED combines information from left ventricular structural pattern (wall thickness and internal cavity size), systolic pressure and myocardial strain and calculates the work done (mechanical energy produced) per unit volume of myocardium during systole. GLASED should prove to be more accurate than the ejection fraction and myocardial strain in assessing left ventricular myocardial function.

## Materials and methods

### Subjects

We investigated 183 subjects, of whom 39 were healthy controls: 55 had resistant hypertension, 53 had idiopathic DCM, and 36 had probable cardiac amyloidosis. We have previously published data on this population^16,17^. The demographic details are shown in Table 2. Our normal cohort consisted of normotensive patients with no history of hypertension or cardiac disease, a normal ECG, and were on no regular medication. Hypertensive subjects were recruited from a tertiary hypertension clinic. The DCM cohort was taken from our CMR database between January 2013 and January 2015 and had no clinical or radiological evidence of ischemic heart disease such as regional late gadolinium enhancement. The cardiac amyloid patients were diagnosed using clinical features with a typical CMR phenotype including late gadolinium enhancement pattern or biopsy when this was available.

**Table 2:** Demographic findings and results.

Results	Normal	Hypertension	Dilated cardiomyopathy	Amyloid
Number (M/F)	39 (21/18)	55 (32/23)	53 (37/16)	36 (32/4)
Age (years)	45 ± 14	52 ± 13*	54 ± 16*	70 ± 10***^†††‡‡‡^
BMI (kg/m^2^)	25.7 ± 4.7	30.5 ± 4.3***	28.5 ± 6.2^†^	25.6 ± 3.7^†††^
Weight (kg)	75.2 ± 14.6	90.4 ± 15.5***	85.6 ± 20.9*^†^	76.6 ± 14.8^††^
BSA (m^2)^	1.88 ± 0.21	2.07 ± 0.22**	2.02 ± 0.27*	1.91 ± 0.21^††^
Systolic BP (mmHg)	125 ± 12	174 ± 29***	116 ± 16^†††^	133 ± 29^†††‡‡^
Diastolic BP (mmHg)	76.3 ± 9.1	99 ± 16***	68 ± 14**^†††^	83 ± 10*^†††‡‡‡^
LVEDV (mL)	146 ± 40.0	177 ± 34.2*	267 ± 59.2***^†††^	164 ± 67.0^‡‡‡^
LVESV (mL)	55.4 ± 21.4	61.2 ± 21.5	189 ± 63.6***^†††^	89.7 ± 60.0**^†‡‡‡^
Stroke volume (mL)	90.6 ± 23.7	115.0 ± 23.4***	76.9 ± 27.5*^†††^	74.4 ± 22.6*^†††^
SV/BSA (mL/m^2^)	48.1 ± 11.9	55.5 ± 9.7*	38.5 ± 12.8**^†††^	41.3 ± 18.4^†††^
SV/Height^2.7^ (mL/m^2.7^)	52.7 ± 12.5	66.9 ± 13.7***	44.0 ± 15.0*^†††^	43.0 ± 12.5*^†††^
LV muscle mass (g)	109 ± 29	200 ± 55***	187 ± 48***	215 ± 78***
LVIDd (mm)	52 ± 5.1	51 ± 4.5	67 ± 5.0***^†††^	52 ± 7.4^‡‡‡^
LVIDs (mm)	35.1 ± 4.6	31.6 ± 5.9	59.4 ± 5.9***^†††^	40.6 ± 9.7**^†††‡‡‡^
EDWT (mm)	7.6 ± 1.2	10.5 ± 2.5***	7.9 ± 1^†††^	14.1 ± 2.7***^†††‡‡‡^
ESWT (mm)	12.0 ± 1.6	16.8 ± 3.4***	10.1 ± 1.8**^†††^	17.9 ± 2.8***^‡‡‡^
LV length (mm)	96.7 ± 8.5	99.8 ± 8.0	105 ± 7.9***^††^	98.5 ± 8.3^‡‡^
EF (%)	63.8 ± 6.6	66.0 ± 8.4	29.6 ± 11.2***^†††^	49 ± 15.5***^†††‡‡‡^
EF_c_ (%)	64.8 ± 5.6	59.0 ± 8.8**	35.2 ± 7.6***^†††^	39.6 ± 9.2***^†††^
Myocardial Contr. Frac. (%)	89.9 ± 16.5	63.9 ± 15.4***	45.8 ± 17.6***^†††^	40.8 ± 18.2***^†††^
Long. shortening (%)	15.5 ± 2.5	10.8 ± 2.6***	5.8 ± 2.9***^†††^	6.1 ± 2.6***^†††^
Circ. shortening (%)	18.4 ± 2.2	17.3 ± 3.3	7.8 ± 3.1***^†††^	9.3 ± 3.4***^†††^
Radial thickening (%)	61.3 ± 14.6	61.8 ± 14.4	29.9 ± 10.2***^†††^	30.8 ± 13.4***^†††^
Lamé Long. σ SBP (KPa)	25.1 ± 4.8	25.1 ± 8.5	30.2 ± 6.4*^††^	13.5 ± 4.9***^†††‡‡‡^
Lamé Long. σ MAP (KPa)	18.6 ± 3.8	17.8 ± 5.8	21.7 ± 4.7*^†††^	10.1 ± 4.7***^†††‡‡‡^
Lamé Long. σ DBP (KPa)	15.4 ± 3.3	14.2 ± 4.7	17.6 ± 3.9^††^	8.5 ± 3.2***^†††‡‡‡^
Laplace Long. σ SBP (KPa)	28.7 ± 4.9	29.9 ± 9.1**	33.6 ± 6.5***^†^	17.0 ± 5.2^†††‡‡‡^
Lamé Circ. σ SBP (KPa)	57.0 ± 9.9	58.8 ± 18.3	66.8 ± 13.0**^†^	33.1 ± 10.5***^†††‡‡‡^
Laplace Circ. σ SBP (KPa)	57.5 ± 9.9	59.8 ± 18.2	67.2 ± 12.9**^†^	34.0 ± 10.5***^†††‡‡‡^
Cardiomyocyte σ (KPa)	34.6 ± 7.3	31.9 ± 10.4	39.4 ± 8.6^†††^	19.3 ± 7.2***^†††‡‡‡^
Peak TIN force (N)	203 ± 51.2	286 ± 81.1***	385 ± 74.9***^†††^	257 ± 107*^‡‡‡^
Peak Long. force (N)	26.0 ± 6.2	33.5 ± 9.5***	49.2 ± 8.0***^†††^	32.2 ± 13.0*^‡‡‡^
Peak TIN force/LVM (N/g)	1.89 ± 0.31	1.50 ± 0.48***	2.14 ± 0.44*^†††^	1.28 ± 0.47***^‡‡‡^
Peak Long. force/LVM (N/g)	0.24 ± 0.05	0.18 ± 0.07***	0.27 ± 0.06^†††^	0.16 ± 0.06***^‡‡‡^
Stroke work (J)	1.12 ± 0.32	1.88 ± 0.43***	0.86 ± 0.30**^†††^	0.98 ± 0.32^†††^
Stroke work/LVM (J/Kg)	10.4 ± 2.3	9.9 ± 2.7	4.8 ± 1.8***^†††^	5.1 ± 2.4***^†††^
Stroke work/H^2.7^ (J/m^2.7^)	0.262 ± 0.076	0.439 ± 0.103***	0.192 ± 0.066***^†††^	0.225 ± 0.071^†††^
Pressure-strain loop (mHg%)	1.93 ± 0.36	1.86 ± 0.47	0.67 ± 0.34***^†††^	0.81 ± 0.37***^†††^
GLASED MAP (KJ/m^3^))	1.44 ± 0.37	0.988 ± 0.454***	0.618 ± 0.32***^†††^	0.312 ± 0.19***^†††‡‡‡^
GLASED SBP (KJ/m^3^)	1.94 ± 0.49	1.39 ± 0.65***	0.86 ± 0.45***^†††^	0.42 ± 0.28***^†††‡‡‡^
Laplace SSP SBP (KJ/m^3^)	2.14 ± 0.55	1.67 ± 0.74**	0.99 ± 0.45***^†††^	0.58 ± 0.36***^†††‡^
Laplace SSP MAP (KJ/m^3^)	1.58 ± 0.42	1.18 ± 0.52***	0.710 ± 0.38***^†††^	0.432 ± 0.25***^†††‡‡‡^
GLASE SBP (J)	0.199 ± 0.073	0.240 ± 0.082*	0.146 ± 0.080**^†††^	0.077 ± 0.043***^†††‡‡‡^
GLASE MAP (J)	0.147 ± 0.055	0.17 ± 0.056	0.105 ± 0.057***^†††^	0.057 ± 0.03***^†††‡‡‡^
GLASE SBP/BSA (cJ/m^2^)	10.60 ± 3.78	11.76 ± 4.37	7.20 ± 3.59***^†††^	4.09 ± 2.55***^†††‡‡‡^
GLASE SBP/H^2.7^ (cJ/m^2.7^)	4.65 ± 1.55	5.70 ± 2.31*	3.23 ± 1.59*^†††^	1.77 ± 1.06***^†††‡‡‡^
CASED SBP (KJ/m^3^)	5.26 ± 1.24	5.20 ± 2.08	2.60 ± 1.13***^†††^	1.56 ± 0.78***^†††‡‡^
CASE SBP (J)	0.535 ± 0.175	0.917 ± 0.306***	0.450 ± 0.203^†††^	0.287 ± 0.119***^†††‡‡^
CASE SBP/H^2.7^ (J/m^2.7^)	0.125 ± 0.037	0.217 ± 0.083***	0.101 ± 0.043*^†††^	0.066 ± 0.028***^†††‡‡^

Values expressed as mean ± 1 standard deviation.

*Long.* Longitudinal, *Circ.* midwall circumferential, *EDWT* end-diastolic wall thickness, *ESWT* end-systolic wall thickness, *LVIDd* LV internal diastolic diameter, *SV* stroke volume, *LVIDs* LV internal systolic diameter, *BSA* body surface area, *TIN* total inward net force, *LVM* LV muscle mass, *EF*_*c*_ corrected ejection fraction (5 factor)^17^, *Myocardial Contr. Frac.* Myocardial contraction fraction, *SBP* systolic blood pressure, *MAP* mean arterial blood pressure, *σ* stress, *DBP* diastolic blood pressure, *Lapl.* Laplace, *SSP* stress–strain product, *H* height.

*P < 0.05, **P < 0.001, ***P < 0.0001 cf. control.

^†^Dilated cardiomyopathy or Amyloid vs Hypertension.

^‡^Amyloid vs dilated cardiomyopathy. All other comparisons not significant (*P* > 0.05).

The authors confirm that this study complies with the Declaration of Helsinki and that the Research Ethics Committee at the University of Bristol has approved the research protocol. We obtained informed consent from the subjects (or their legally authorized representative).

### Left ventricular structure

CMR was undertaken as outlined in an earlier study and performed at 1.5 T (Avanto, Siemens, Erlangen, Germany) using a spine coil, an 8-element surface array coil and retrospective ECG triggering^17^. MRI analysis was completed by a single reader, with > 5 years MRI experience, using cvi42 software. LV volumes, mass, and EF were measured blinded to the other results. Changes in LV longitudinal length between end-diastole and end-systole were measured manually to calculate myocardial shortening accurately using the 4-point mitral annular plane systolic excursion relative to LV end-diastolic length from the 4-chamber and 2 chamber views^16,22^. End-diastolic wall thickness (EDWT), end-systolic wall thickness (ESWT) and internal dimensions in diastole and systole were measured in the middle of each of the basal and mid (equatorial) LV myocardial segments on the long-axis cines perpendicular to the LV wall whilst allowing for effects of through plane motion. Papillary muscles and trabeculae were excluded from the measurements. Each of these measurements was made 3 times and the means were calculated. Reproducibility (intraclass correlation) for these measurements was excellent (see Appendix). The myocardial contraction fraction was calculated by dividing the stroke volume by the muscle volume.

### Corrected ejection fraction

The EF was adjusted to remove the confounding consequences of abnormalities of mural thickness and internal dimension to give the EF_c_, using a five-variable regression equation derived from the whole study group (Appendix, Eq. 1)^17^.

### Myocardial deformation

Normal strain reference ranges vary between modalities and vendors in part because of differing spatio-temporal smoothing algorithms and type of strain used. Automated methods may also lack validation, comparison and standardisation potentially resulting in inaccuracies. Therefore, long-axis shortening (LAS) was derived using the benchmark of direct measurement rather than estimated using potentially less reliable built-in software.

There is a significant difference in epicardial, midwall and endocardial circumferential strains (~ 3%, 18% and 35%, respectively in normal ventricles), giving a large shear strain gradient, and making it difficult to quote a single value for circumferential strain. To compensate for the strain gradient, we used a standard analytic method to obtain the midwall circumferential shortening (Appendix, Eq. 2)^23^. Proportional radial thickening was calculated as the absolute mural thickening divided by the end-diastolic mural thickness and expressed as a percentage.

### Myocardial stresses and forces

Myocardial stresses were calculated using both the Lamé equations (Appendix, Eq. 3 and 4) and by Laplace’s method (Appendix, Eq. 5) for longitudinal and midwall circumferential stresses. Lamé equations are more accurate for a thick-walled chamber compared to the simpler Laplace’s method devised for thin-walled vessels. Longitudinal force generation was calculated from the longitudinal stress and the mean circumferential cross-sectional areas at the base and equatorial regions. We defined the total inward net (TIN) force during systole as the total scalar normal force exerted on the intraventricular blood that produces the peak ventricular pressure. We calculated the TIN force as the product of systolic pressure and internal surface area of a closed hemi-prolate spheroid.

### Cardiomyocyte contractile stress

To calculate cardiomyocyte orientated stresses on various transmural myocardial levels, we modelled the LV as consisting of ten myocardial shells of equal thickness. The helical angle of each shell was based on an earlier study^24^. The helical angle, end-diastolic diameter, mural thickness, length, and systolic pressures, were used to calculate longitudinal stress and force for each shell (Appendix, Eq. 6). The accumulative total longitudinal force was calculated using numerical integration to give the sum of the myocardial forces across the wall. The cardiomyocyte contractile stress was adjusted to give the estimated total peak longitudinal force for each individual.

### Stroke work and pressure-strain loop

Stroke work was calculated from the mean arterial pressure and stroke volume (Appendix, Eq. 7a). Stroke work was indexed to myocardial weight from the LV muscle volume by assuming a myocardial density of 1.04 g/mL. Quantification of the ventricular work index using global left pressure–strain loop was performed (Appendix, Eq. 7b)^25^.

### Active strain energy and strain energy density

GLASED was calculated using the Lamé derived stress and longitudinal shortening (Appendix, Eq. 8) based on the stress–strain relationship (see Appendix). Similar calculations were made for midwall circumferential active strain energy density (CASED) (Appendix, Eq. 9). For completeness, we calculated GLASED and CASED using both peak systolic blood pressure and mean arterial pressure. CASE and GLASE were derived by multiplying their respective ASED by the LV muscle volume. CASE and GLASE were indexed to body surface area (BSA) and height^2.7^.

The Laplace stress–strain product (SSP) was calculated using the longitudinal Laplace wall stress calculation and longitudinal shortening (Appendix, Eq. 10).

### Statistical analyses

Our primary, a priori hypothesis was that ASED (GLASED and GLASE) would differ in the four cohorts. We assessed normality using the Shapiro–Wilk Test. Means were compared between all cohorts using one-way ANOVA with Tukey HSD/Kramer test or Kruskal–Wallis test with Nemenyi test as appropriate to correct for multiple comparisons. Correlations were performed using Pearson’s method. All continuous variables are displayed in Table 2. A *P* < 0.05 was considered statistically significant.

## Results

**Figure 1:**
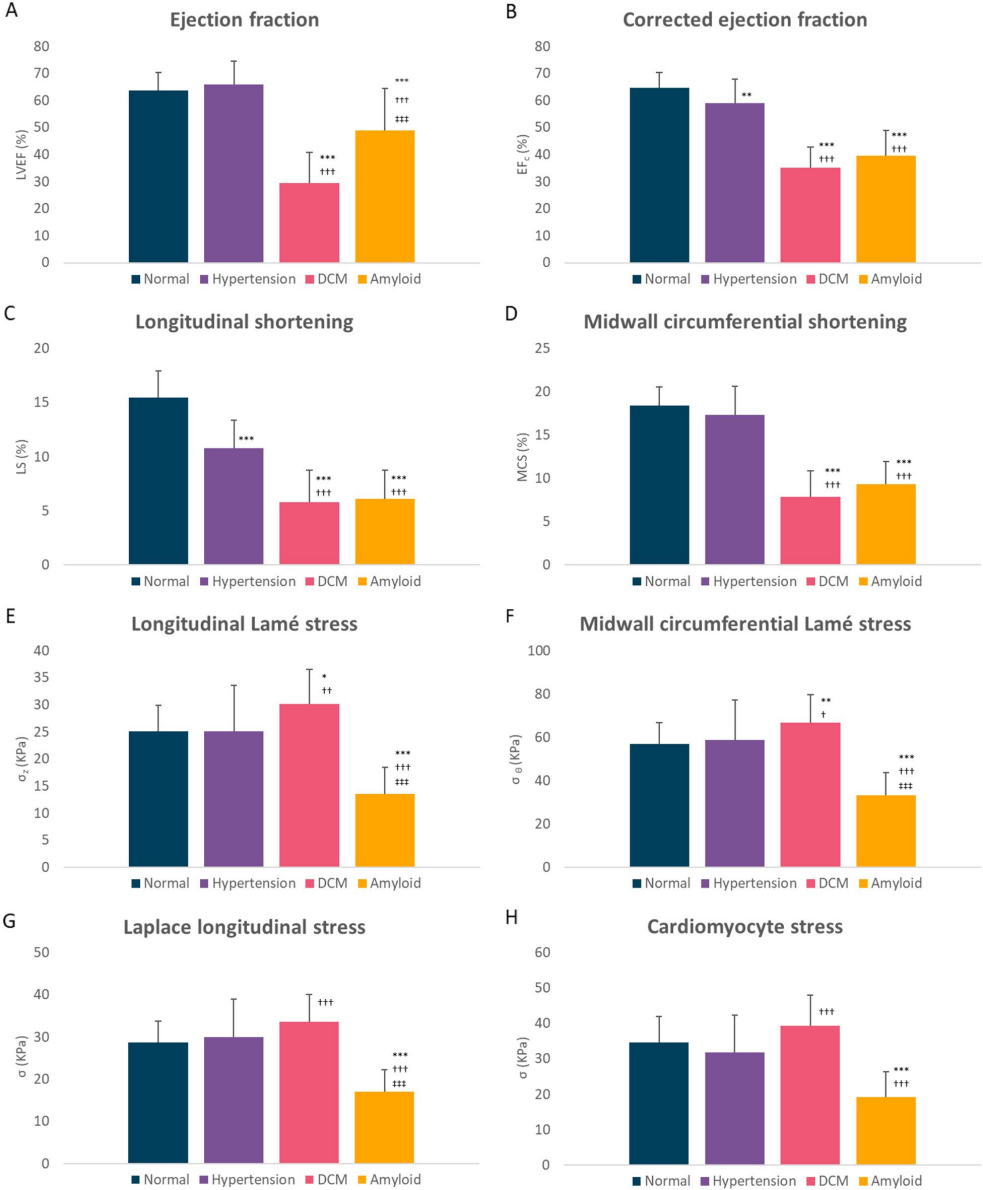

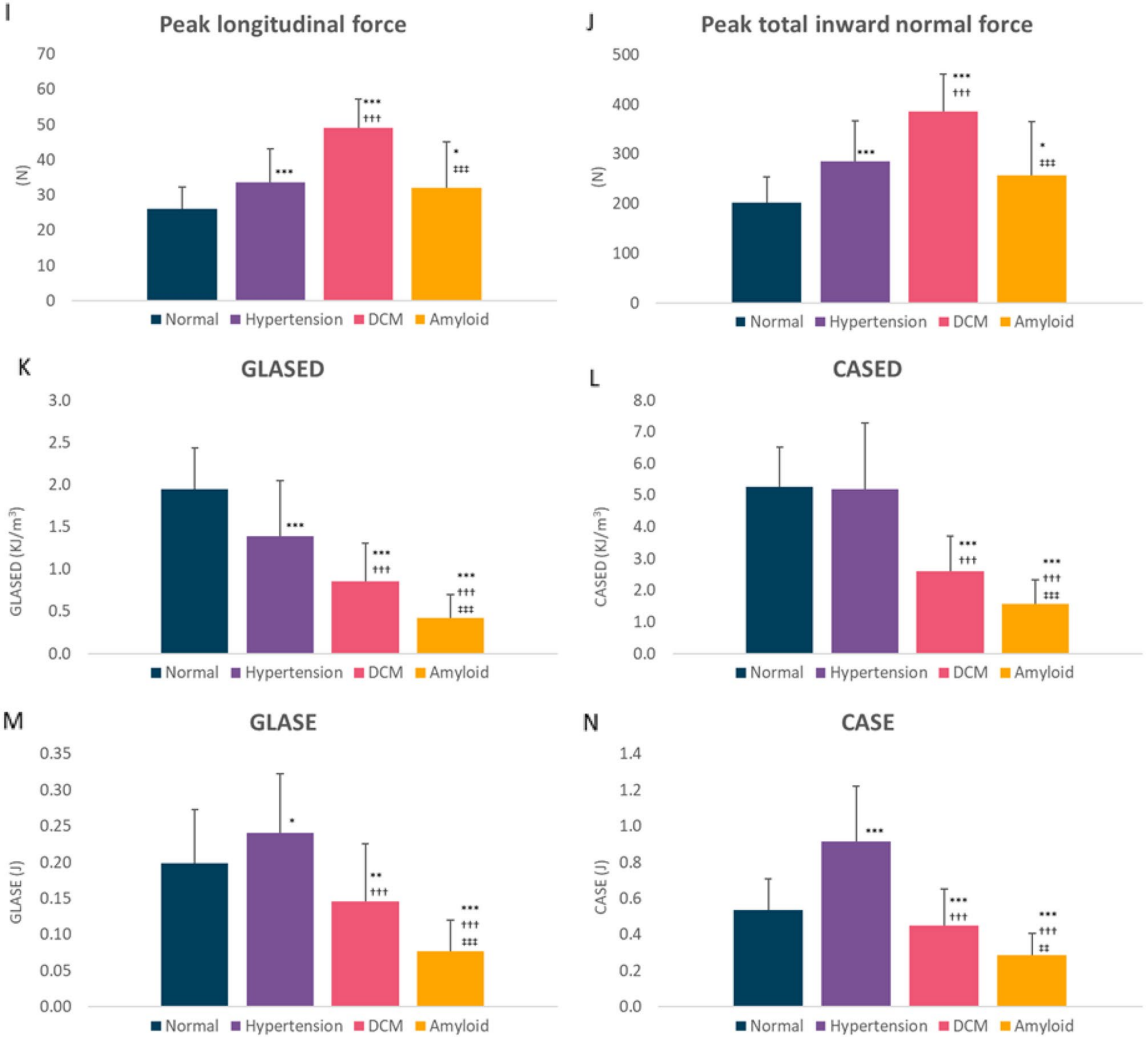
Graphs showing main results. Global longitudinal active strain energy density (GLASED), midwall circumferential strain energy density (CASED), global longitudinal active strain energy (GLASE) and midwall circumferential strain energy (CASE) (mean ± SD). *P < 0.05, **P < 0.001, ***P < 0.0001 cf. control. ^†^Dilated cardiomyopathy or Amyloid vs Hypertension. ^‡^Amyloid vs Dilated cardiomyopathy. All other comparisons not significant (*P* > 0.05).

**Figure 2:**
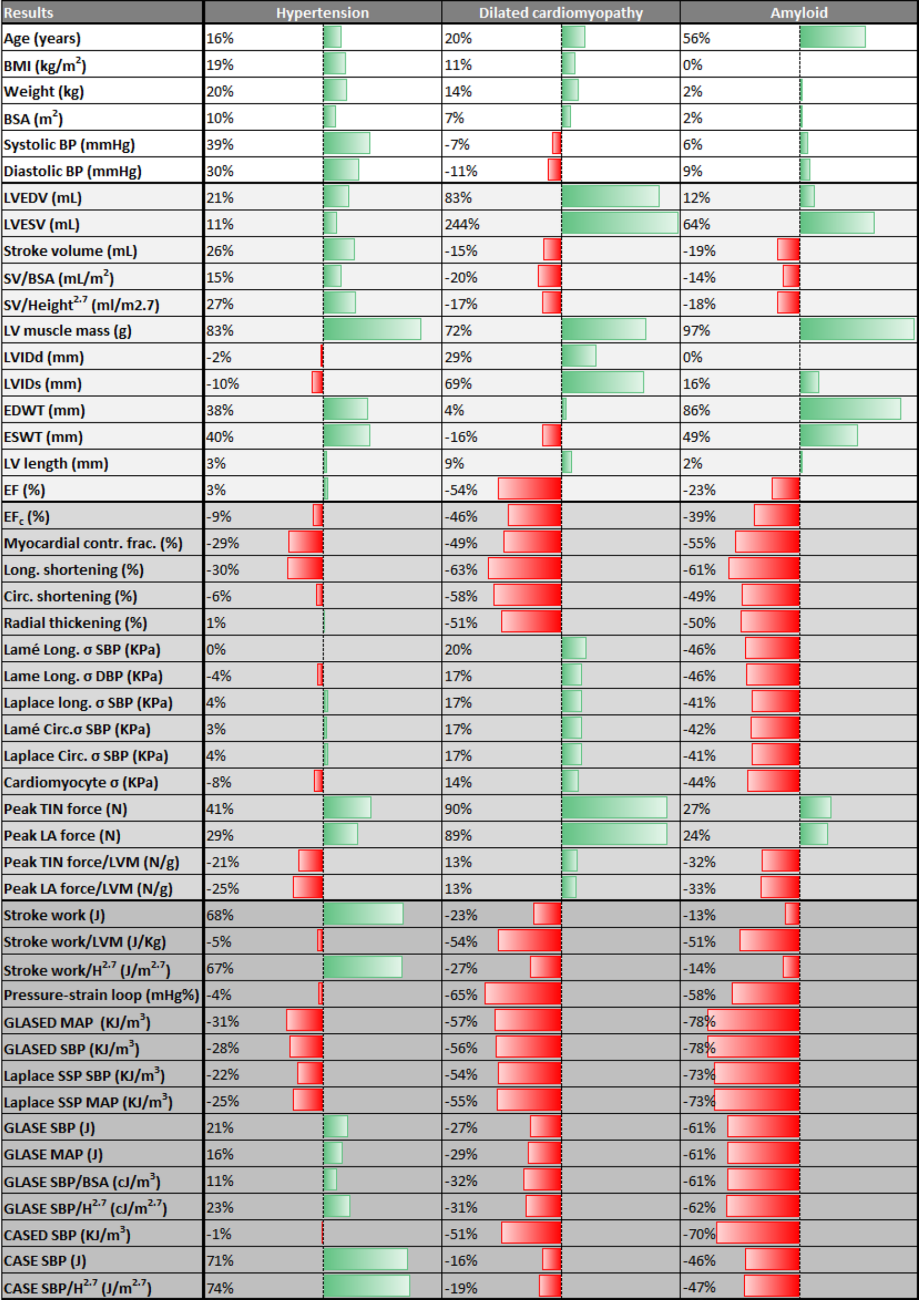
Percentage changes compared with control cohort. Values as a percentage of normal cohort with data bars (range ± 100%). Note that LV end-systolic volume for dilated cardiomyopathy cohort is truncated at + 100%. Abbreviations as in Table 2.

Detailed results are shown in Table 2. Column graphs of the main results are presented in Fig. 1 and relative proportional changes compared with control are shown in Fig. 2. The mean age of the amyloid cohort was higher and the mean BMI was greater in the hypertension cohort. The mean blood pressure was lowest in the DCM cohort.

Stroke volume was significantly increased in the hypertension cohort, but significantly decreased in DCM and amyloid when compared with controls. The LV muscle mass was increased in hypertension, DCM, and amyloid. End-diastolic wall thickness was increased in hypertension and amyloid but was unchanged in DCM. The mean EF was not significantly different in the controls compared with hypertension but was decreased in the DCM and in amyloid (Fig. 1A). The myocardial contraction fraction was significantly reduced in hypertension, DCM and amyloid but there was no difference between the amyloid and DCM cohorts.

The EF_c_ was significantly reduced in amyloid, but this was not significantly different from the DCM cohort (Fig. 1B). The EF_c_ was significantly higher in DCM, and lower in amyloid when compared with the standard EF.

Longitudinal shortening was reduced in hypertension, DCM and in amyloid when compared with the normal cohort (Fig. 1C). Midwall circumferential shortening was unchanged in hypertension but significantly decreased in DCM and amyloid (Fig. 1D). In contrast, proportional radial thickening was unchanged in hypertension but decreased in DCM and amyloid.

Lamé longitudinal stress was unchanged in hypertension, significantly increased in DCM, but decreased in amyloid (Fig. 1E). Midwall circumferential stress was unchanged in hypertension, increased in DCM, but decreased in amyloidosis (Fig. 1F). Laplace longitudinal stress was unchanged in hypertension, increased in DCM and decreased in the amyloid cohort (Fig. 1G). Cardiomyocyte contractile stress was unchanged in hypertension and DCM compared with control but was decreased in amyloid (Fig. 1H).

Peak longitudinal force was higher in hypertension, DCM and amyloid (Fig. 1I). Total inward net force was also higher in hypertension, DCM and amyloid (Fig. 1J). TIN force per unit LV muscle mass and peak longitudinal forces per LV muscle mass were decreased in hypertension and amyloid but increased in DCM.

Stroke work derived from the pressure–volume loop was significantly increased in hypertension and decreased in DCM, but no different in amyloid. Stroke work per LV muscle mass was reduced in DCM and amyloid but did not differ between the normal and hypertension cohorts or between DCM and amyloid. LV pressure-strain loop was unchanged in hypertension compared with the control group but was decreased to the same degree in DCM and amyloid.

**Figure 3:**
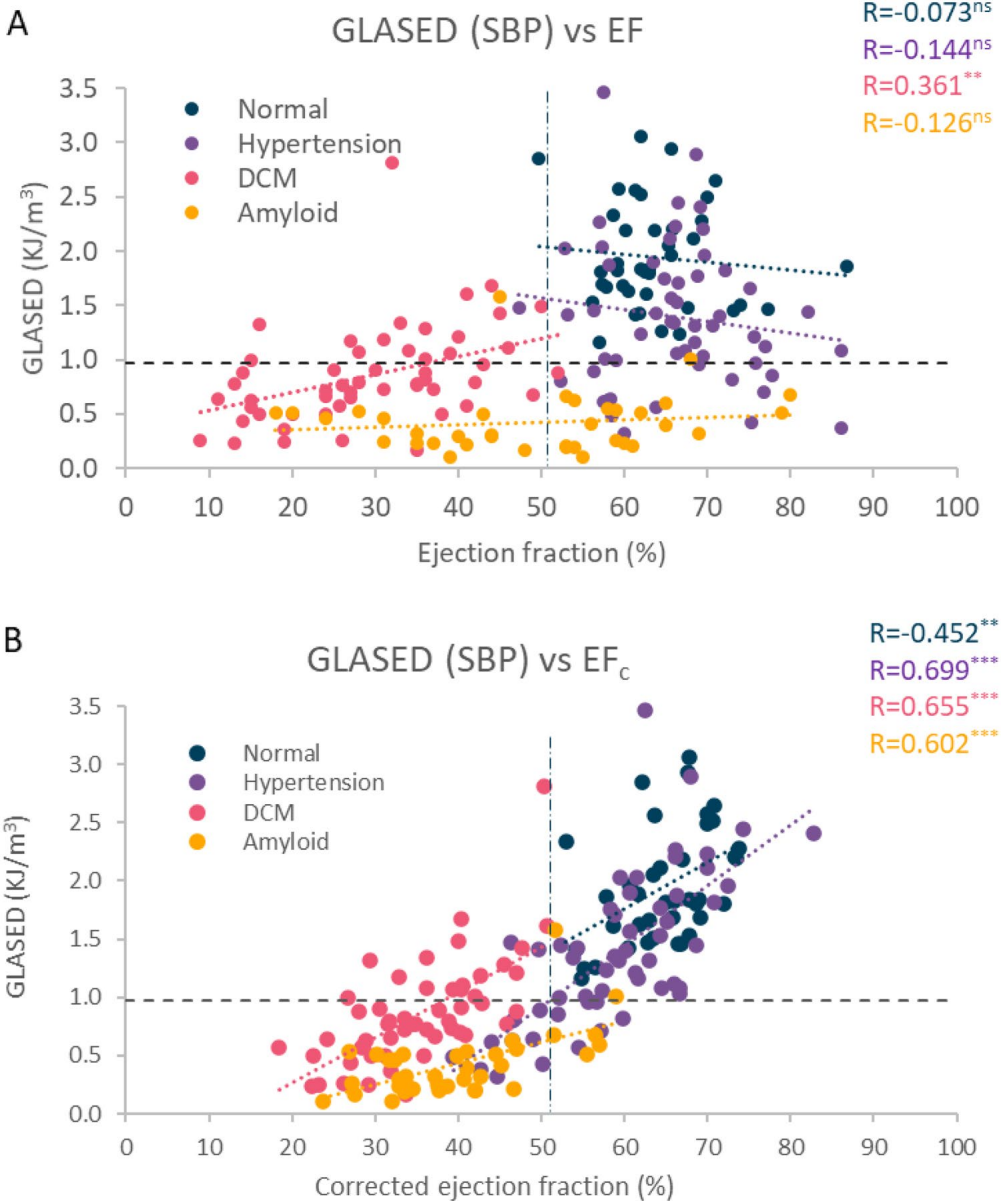
Scatter plot showing ejection fraction (**A**) and corrected ejection fraction vs GLASED (**B**). Dashed lines show the lower end of the 95% reference range for the normal cohort giving a cut-point of GLASED of 0.968 kJ/m^3^ and for EF of 51%.

GLASE was higher in the hypertension cohort, but lower in DCM and amyloid (Fig. 1M). GLASE indexed to BSA and height^2.7^ showed similar changes (Fig. 2). GLASED was reduced progressively in hypertension, DCM and amyloid (Fig. 1K). The 95% reference range in the control group gave a GLASED cut-point of 0.968 kJ/m^3^ (Fig. 3). CASED was unchanged in hypertension and reduced in DCM and in amyloid (Fig. 1L).

CASE was increased in the hypertension cohort (Fig. 1N). Comparable trends were found when mean arterial pressure was used for both GLASED and GLASE, but with lower values.

Laplace SSP calculated using peak systolic pressure was reduced in hypertension, DCM and amyloid. We have summarised our key findings in Table 3.

**Table 3:** Summary of different methods of assessing LV performance in the three disease cohorts compared to control.

Method	Hypertension	DCM	Amyloid
EF	–	↓↓↓	↓
EF_c_	↓	↓↓	↓↓
Pressure-strain loop	–	↓↓↓	↓↓↓
CASE	↑↑↑	–	↓↓
GLASE	↑	↓	↓↓
CASED	–	↓↓↓	↓↓↓↓
GLASED	↓	↓↓↓	↓↓↓↓
Expected survival	↓	↓↓↓	↓↓↓↓

Note that GLASED distinguishes the four cohorts and corresponds to the expected survival in the disease cohorts compared to controls.

## Discussion

Though there are many measures available to characterise the performance of LV, to the best of our knowledge, this is the first study to report a comprehensive comparison of measures of LV systolic performance that includes strain energy density in patients with hypertension, DCM, and amyloid heart disease. GLASED provides 4 important advantages over most previous methods. GLASED is: (i) based on sound engineering principles; (ii) calculates the fundamental measure of energy production (i.e. work done) per unit volume of myocardium; (iii) accounts for the known confounding variables such as haemodynamic load and ventricular remodelling; (iv) matches the anticipated symptom severity, brain natriuretic peptide (BNP) and mortality in our cohorts.

The synopsis of results for each cohort was as follows:

### Hypertension

The hypertension cohort had a normal EF, a mildly reduced EF_c_, a moderately reduced myocardial contraction fraction and longitudinal strain with a normal longitudinal stress. The greater stroke volume/height^2.7^ was consistent with a high-output state. Both the TIN and longitudinal generated forces were moderately increased. Pressure-strain loop was unchanged. Stroke work and CASE were moderately increased, and GLASE was mildly elevated, consistent with the extra work needed to generate the elevated blood pressure. A significant reduction in GLASED would suggest early hypertensive heart disease that was driven by the fall in longitudinal shortening of 30%. CASED, however, was not reduced as midwall circumferential shortening did not fall significantly. The explanation for difference between CASED and GLASED is uncertain but may reflect subendocardial ischaemia in the hypertension cohort due to microvascular disease.

### Dilated cardiomyopathy

The EF, EF_c_, myocardial contraction fraction, longitudinal and midwall shortening were all severely reduced in the DCM cohort. The cohort also had a normal mural thickness, with remarkably high longitudinal and TIN force production. The EF_c_ was higher than the EF. All the stresses in dilated cardiomyopathy were higher than healthy controls. Stroke work, pressure-strain loop, GLASE, GLASED, CASE and CASED were all moderately reduced, consistent with both a reduced LV energy production and reduced energy production per unit muscle volume.

### Amyloid heart disease

The EF was mildly reduced in the amyloid cohort, but the EF_c_, myocardial shortening, proportional radial thickening and all the stresses were severely decreased. The peak longitudinal force and TIN force were increased compared to the control group. Myocardial contraction fraction in the amyloid and DCM cohorts were the same. Stroke work per myocardial mass was also markedly decreased. Pressure-strain loop was reduced to a similar degree as DCM. In contrast, the GLASE, GLASED, CASE, CASED and SSP were extremely low. The amyloid cohort had the lowest ASE and ASED of the four groups, suggesting the worst systolic dysfunction and poorest contractile function. This suggests that the notion of amyloid heart disease being the archetypal form of diastolic dysfunction with relatively preserved “systolic function”, as defined by EF, needs re-evaluation.

### Strain energy density vs alternative methods

The EF_c_ was better at quantifying abnormalities compared with the EF. GLASED provided a sensitive means of detecting mild hypertensive heart disease and was different between the amyloid and DCM cohorts. A finding that contrasted with the alternative methods. Clear distinctions existed between the EF and GLASED in the four cohorts. The EF is emergent from, and contingent upon, known variables (Table 1)^10–12,14,16–18^. ASED the same input variables as EF, namely myocardial strain, mural thickness, and internal dimensions but also includes pressure-generation information. A decrease in longitudinal or midwall shortening decreases EF, EF_c_, GLASED and CASED (Table 1). In contrast, increasing LV diameter decreases EF but raises ASED. A greater mural thickness increases EF (without an improvement in pump performance) but decreases ASED (Table 1).

It can be appreciated why EF is not an ideal method of measuring LV performance since it does not allow for either the effect of haemodynamic load or geometric changes. The EF, under high-loading conditions, is maintained because of the adaptation of the myocardium and the structural changes such as parallel cardiomyocyte hypertrophy. Of the thirty-two individuals with a preserved EF, just under one-fifth of our overall cohort, had significantly reduced GLASED values. This suggests that the use of EF would miss a sizeable proportion of patients with important myocardial dysfunction. In contrast, the EF_c_, by removing the confounding effects of LV mural thickness and dimensions^17,19^, only misses nine individuals, or one-twentieth, of those with a reduced GLASED.

Work performed by the left ventricle was calculated using both the stroke work (pressure–volume loop) and the pressure-strain loop methods^25^. Neither of these techniques distinguish between normal and hypertension or between DCM and amyloid because they do not correct for wall thickness or ventricular size by calculating wall stress.

### Myocardial forces, stresses and strains

Myocardial forces such as TIN and longitudinal forces were increased in each of the disease cohorts especially so in DCM. Correcting these forces for LV mass improved their utility although they remained high in DCM. Using myocardial stress alone missed the abnormalities in hypertension and was misleading in assessing the contractile abnormalities of DCM. Myocardial strains were reduced in all the disease cohorts apart from midwall shortening in the hypertensive cohort. Importantly, myocardial strains alone did not differentiate amyloid from DCM.

### GLASED vs Laplace stress–strain product

The GLASED values provide a more exact assessment for ASED compared with SSP, since it uses the more appropriate Lamé equation to calculate the stress for a thick-walled chamber. The simpler SSP gave comparable results, albeit with a slight underestimate in the amyloid cohort and an overestimate in the DCM cohort. SSP, however, is an alternative with a simpler equation to use.

### Symptoms and prognosis

**Figure 4:**
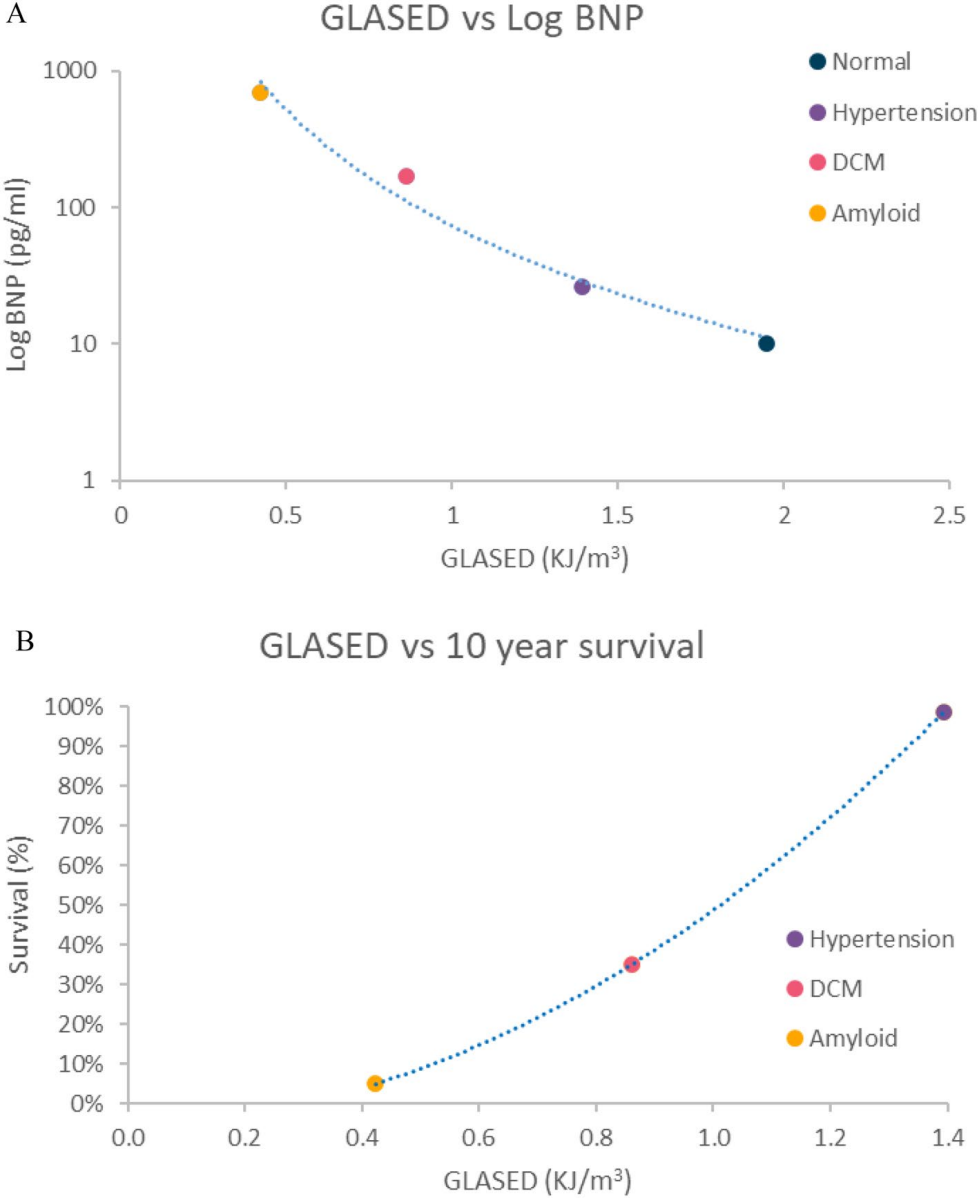
Plots showing expected relationship between GLASED and BNP and 10-year survival based on previously published data. (**A**) BNP provides important prognostic information and correlates with symptoms. Graph shows the potential association between GLASED and BNP (not age or sex matched)^25,26^. High levels of BNP appear to be linked with low levels of GLASED. (**B**) Association between GLASED and expected mortality^27–29^. Low levels of GLASED may be coupled with an extremely poor survival.

We did not assess either the severity of symptoms, BNP or prognosis in our cohorts. The reduced indexed stroke volume in the amyloid and DCM cohorts would suggest our patients had mild low-output states and heart failure. Symptom severity is usually marked in amyloid heart disease, moderate in treated DCM, minor or absent in hypertensive heart disease. Plasma BNP levels are correlated with both symptoms and prognosis. BNP is highest in amyloid heart disease (~ 690 pg/mL), followed by DCM (~ 170 pg/mL), hypertension (~ 26 pg/mL) and normal individuals (~ 10 pg/mL)^26,27^. This trend is reflected by our GLASED data (Fig. 4A). The expected 10-year mortality in amyloid heart disease, DCM, and hypertension is 95%, 65% and 1.5% respectively (Fig. 4B)^28–30^. Reduced myocardial strain^4,31,32^, higher internal diastolic diameter^33^, low blood pressure in heart failure^34^ and concentric LV hypertrophy (i.e. increased wall thickness)^35,36^ are individually associated with an increased risk, and each of these risk factors reduces GLASED (Table 1), indicating that GLASED has a highly promising role in risk assessment.

### Implications for heart failure

A key physiological requirement of the heart is to maintain adequate tissue perfusion both at rest and with exercise, even in the presence of myocardial disease^37–39^. The left ventricle achieves this through remodelling^37–39^. Tissue and organ perfusion is, however, independent of the EF, since resting stroke volume and cardiac output are often preserved in heart failure^39^. There is a high incidence of hypertension, concentric hypertrophy, reduced wall stress and reduced myocardial strain in heart failure with a preserved EF^40^. Assessment of suspected HFpEF patients using GLASED would provide additional insight to the complicated mechanisms of this enigmatic and heterogenous condition by establishing the contribution of myocardial contractile abnormalities in HFpEF subgroups.

### Myocardial function and contractility

The phrase “myocardial contractility” is used ubiquitously in both clinical and experimental studies. Despite this widespread use, contractility remains ill-defined 125 years after being introduced. GLASED provides a quantifiable definition of myocardial function by combining its known features, namely force generation, active stress, and deformation. Furthermore, changes in contractile function so defined would allow for the definition of inotropy to be clarified and, importantly, quantified. To date, there are no current measures of contractility that are applicable to in vitro, ex vivo and in vivo studies. Myocardial ASED, however, is applicable to both types and provides a method to compare in vivo and in vitro studies^41^. We recently introduced the term contractance as a measure of myocardial function, which is defined and quantified by myocardial ASED^41^. The definitions of ventricular performance and myocardial function are simplified and unified by the term contractance. We suggest that myocardial ASED could be used in clinical practice to assess the health of the myocardial muscle, since it identifies myocardial systolic and contractile dysfunction more accurately than current methods.

### Limitations

Our cohorts were of limited size and were not age or sex matched as our aim was to explore distinct disease cohorts with different, yet near symmetrical, LV geometric patterns. Details of their cardioactive medication were not available. We did not use the current recommended criteria for diagnosis of amyloid as this was a historical cohort. These limitations would not alter the conclusions of this study as the analyses are centred around the structural and haemodynamic changes. Indeed, we think that this is a strength of the methods used as they consider the structural changes that may occur with age, sex or previous hypertension as well as the haemodynamic effects of any drug therapy. We were unable to provide information from the time-volume curves as the software necessary was unavailable when the study was performed. Further studies will be required to validate our findings.

Numerical methods derived from an anisotropic visco-hyperelastic non-linear constitutive model could provide more exact results but would only be possible in a research environment and performed on a small number of cases using methods such as finite element modelling^42,43^. The calculations for ASE and ASED ignored the small amount of work performed in deforming myocardium alone and the minor component of kinetic energy imparted on the ejected blood’s motion. This is also a limitation of other methods such as stroke work calculations. Our study lacks external validation and direct prognostic data although the indirect evidence is highly promising (Fig. 4A,B). Future studies looking at GLASED potential role in assessing prognosis are required prior to widespread implementation outside of a research environment. Calculation of ASED may be prone to propagation error as three of the terms are squared. This emphasises the need for accurate measurements of the input variables in any future studies. Though we make these limitations explicit, however, we believe that those limitations do not affect our conclusion on the potential advantage of GLASED as a promising new marker of the LV performance.

## Conclusions

An accurate assessment of left ventricular systolic function is essential for both risk evaluation and management decisions in most cardiac diseases. However, the EF and myocardial strain data miss valuable information concerning myocardial contractility. The new measure, GLASED, provides a quantifiable pathophysiological assessment of intrinsic myocardial function. Furthermore, GLASED closely matches the expected symptom severity, BNP, and mortality in these conditions. GLASED combines information from both stress and strain and estimates the longitudinal work done (mechanical energy) per unit volume of myocardium, and, therefore, gives important mechanistic insight into the pathophysiology of heart failure syndromes and has the potential to provide prognostic information.

## Supplementary Information

Supplementary Information 1.

Supplementary Information 2.

## Data Availability

The corresponding author will share the data underlying this article on reasonable request.

## Abbreviations

ASE: Active strain energy
ASED: Active strain energy density
BNP: Brain natriuretic peptide
BSA: Body surface area
CASE: Midwall circumferential active strain energy
CASED: Midwall circumferential active strain energy density
EF: Left ventricular ejection fraction
EF_c_: Corrected ejection fraction
LV: Left ventricle/ventricular
GLASE: Global longitudinal active strain energy
GLASED: Global longitudinal active strain energy density
SSP: Laplace stress–strain product
