# Supplementary material for: Left ventricular active strain energy density is a promising new measure of systolic function
Source: Sci Rep. 2022 Jul 26;12:12717. doi: 10.1038/s41598-022-15509-8 (PMC9325776; doi:10.1038/s41598-022-15509-8)
Supplement: Supplementary file 2 — Supplementary Information 2. [file 41598_2022_15509_MOESM2_ESM.pdf]

Left ventricular active strain energy density is a promising new measure of systolic function

Appendix

Corrected ejection fraction

The EFc was calculated using the following regression equation[17]:

EF_c (%) = 38.9-0.423D+2.07W-0.648ε_z -2.07ε_θ -0.174L (R^2 = 0.856, P<0.0001).

(Eq 1)

Where D = LVIDd (mm), W=EDWT (mm), ε_z = longitudinal shortening, ε_θ = midwall circumferential shortening. To calculate the EF_c, we inputted D and W using the mean of the normal control cohort i.e. D = 52 mm, W = 7.6 mm and the longitudinal shortening (ε_z), midwall circumferential shortening (ε_θ) and length (L) were inputted as the measured values in each individual.

Midwall circumferential shortening

Midwall circumferential shortening (ε_θ) was estimated using the following equation:

$$\varepsilon_\theta\ (\%) = \frac{((\mathrm{LVIDd} + \mathrm{EDWT}) - (\mathrm{LVIDs} + \mathrm{H}))}{(\mathrm{LVIDd} + \mathrm{EDWT}) \times 100} \qquad \text{(Eq 2)}$$

where H = $((\mathrm{LVIDd} + \mathrm{EDWT})^3 - (\mathrm{LVIDd})^3 + (\mathrm{LVIDs})^3)^{1/3}$ - LVIDs

Stresses

Longitudinal Lamé stress

$\sigma_z = \frac{P_i r_i^2 - P_o r_o^2}{(r_o^2 - r_i^2)}$ and since pericardial pressure is small the $P_o r_o^2$ term can be ignored, and the equation simplified to:

$$\sigma_z = \frac{P_i r_i^2}{(r_o^2 - r_i^2)} \qquad \text{(Eq 3)}$$

Midwall circumferential (hoop) Lamé stress

$$\sigma_\theta = \frac{P_i r_i^2 - P_o r_o^2}{(r_o^2 - r_i^2)} - \frac{r_i^2 r_o^2 (P_o - P_i)}{r_m^2 (r_o^2 - r_i^2)} \qquad \text{(Eq 4)}$$

where P_i is inner (ventricular cavity) pressure (in Pa), P_o is outer (pericardial) pressure and r_m is midwall, r_o is outer (epicardial), and r_i is inner (luminal) LV radii. We assumed P_o to be atmospheric, i.e., *P_o = 0 - gauge pressure*).

Laplace longitudinal stress

$$\text{Laplace longitudianal stress} = P \times \frac{D}{4t}, \qquad \text{(Eq 5)}$$

where P is systolic pressure, *D* is internal diameter, *t* is mean wall thickness.

Cardiomyocyte stress

Longitudinal force per shell=cos(θ) × cardiomyocyte stress × π(S^2+2rS)   (Eq 6)

where r=inner edge of shell radius, S = shell thickness, θ = helical angle in radians.

Assuming a 10-shell model S=t/10 where t=end-diastolic wall thickness. Cardiomyocyte stress is then calculated so that total accumulative force for all the shells, calculated using numerical integration, is the same as that determined from longitudinal stress using the Lamé equation (Eq 3).

Stroke work

SW = MAP × SV   (Eq 7a)

where MAP = (SBP + 2 × DBP)/3. SBP is peak systolic blood pressure and DBP is diastolic blood pressure measure in Pa.

LV pressure-strain loop (LV PSL) index

$\text{LV PSL} = \text{SBP} \times \varepsilon_z(\%)$ in mmHg%   (Eq 7b)

converted from mmHg% to mHg% by dividing by 1,000.

Strain energy density and strain energy

GLASED was calculated using the longitudinal Lamé derived stress (σ_z) and longitudinal shortening (ε_z), *such that:*

GLASED = ½ × σ_z × ε_z .   (Eq 8)

CASED was calculated using the midwall circumferential (hoop) Lamé derived stress (σ_θ) and midwall circumferential shortening (ε_θ), *such that:*

CASED = ½ × σ_θ × ε_θ (Eq 9)

GLASE and CASE were calculated from M × GLASED and M × CASED respectively, where M= left ventricular muscle volume derived by MRI.

The Laplace stress-strain product (SSP)

Laplace longitudinal stress strain product was calculated as follows:

$$SSP = ½ * \varepsilon_z \times Laplace\ stress$$

$$= \varepsilon_z \times P \times \frac{D}{8t}, \qquad \text{(Eq 10)}$$

where ε_z is longitudinal shortening, *P* is systolic pressure, *D* is internal diameter, *t* is mean wall thickness.

Worked example showing the calculation of GLASED

$$\text{GLASED} = \tfrac{1}{2} \times \sigma_z \times \varepsilon_z$$

$$= \tfrac{1}{2} \times \frac{P_i r_i^2}{(r_o^2 - r_i^2)} \times \varepsilon_z$$

Let $P_i = 16{,}665.3$ Pa (SBP 125 mmHg), $r_i = 0.26$ m (half of LVIDD = 52 mm), $r_{o=}\ 0.336\ m\ (r_i + EDWT = 26 + 7.6\ mm)$ and $\varepsilon_z = 15.2\%$

$$= \tfrac{1}{2} \times \frac{16{,}665 \times 26.0^2}{(33.6^2 - 26.0^2)} \times 15.2\%$$

$$= \tfrac{1}{2} \times \frac{16{,}665 \times 676}{(11{,}29 - 676)} \times 15.2\%$$

$$= \tfrac{1}{2} \times \frac{11{,}265{,}540}{453} \times 15.2\%$$

$$= \tfrac{1}{2} \times 24{,}871 \times 15.2\%$$

GLASED = 1.93 KJ/m^3

Active strain energy density: comparison of non-linear and linear methods

A typical pressure-strain curve was derived from a study by Loncaric and colleagues (Figure A1).[44] The curve was divided into 13 points adjusting mural thickness and ventricular diameter between mitral valve closure (MVC) and aortic valve closure (AVC) and a stress-strain curve plotted (Figure A2). The individual longitudinal stress for each of the 12 periods were calculated using the Lamé equation and the longitudinal ASED (GLASED) was calculated from the sum of the ASED for each period and giving the area under the curve between MVC and AVC (Figure A3).

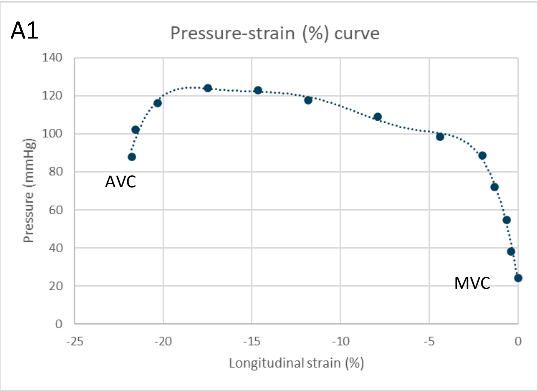

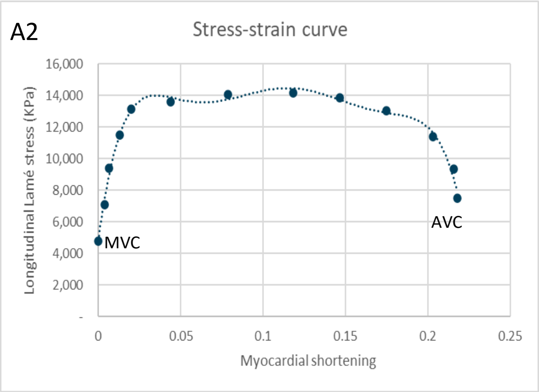

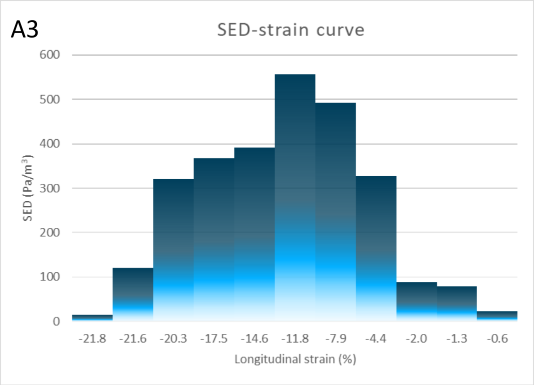

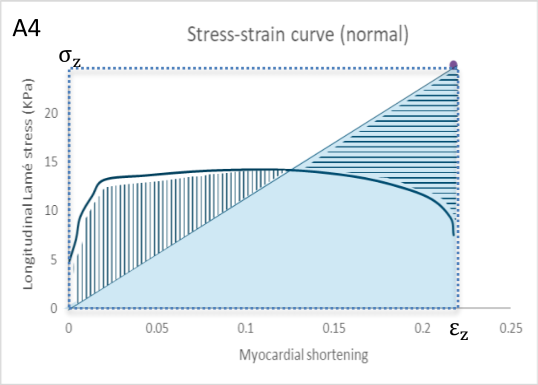

In this example, the GLASED was 2.80 KPa/m^3 (non-linear method) and using Eq 8 was 2.71 KPa/m^3 (linear method - blue triangle A4) suggesting that using the ½ factor is a reasonable compromise for routine use in clinical practice. Geometrically, the ½ factor is half the area of the rectangle with height (σ_z, *peak stress based on SBP)* and width (ε_z, *peak strain)* with area of ($\sigma_z \times \varepsilon_z$, *dashed box*). Note that this compromise is achieved because the vertical hatched area is similar to the horizontal hatched area in Figure A4.

A downloadable Excel spreadsheet to calculate MASED is available online.

Strain energy density proof

SED can be derived from the definition of Work i.e. $F \times l$

where F = force, l = displacement/distance, V=volume, A = cross sectional area, L, original length.

$Energy\ (work) = ½ \times F \times l$, where F is the peak force (see Figure A4).

$= ½ \times \frac{F \times l}{V} \times V$, where V is volume.

$= ½ \times \frac{F \times l}{A \times L} \times V$, where A is cross sectional area, L is original length.

$$= ½ \times \frac{F}{A} \times \frac{l}{L} \times V$$

Substituting $\sigma = \frac{F}{A}$ and $\epsilon = \frac{l}{L}$, gives

Strain energy (SE) $= ½ \times \sigma \times \epsilon \times$ V, and

Strain energy density (SED) =*SE/V*

$$= ½ \times \sigma \times \epsilon$$

Reproducibility: Interclass correlation Class 2, Type 1 and Alpha 0.05 in 20 individuals from control group

Measurement	ICC	Lower	Upper
LV ED length 4C	0.984	0.960	0.990
LV ED length 3C	0.983	0.959	0.993
LV ED length 2C	0.990	0.974	1.000
LV ES length 4C	0.991	0.978	0.996
LV ES length 3C	0.990	0.975	0.996
LV ES length 2C	0.984	0.960	0.994
EDWT	0.981	0.952	0.992

ED, end-diastolic; ES, end-systolic; 4C, 4 chamber view; 3C, 3 chamber view; 2C, 2 chamber view; EDWT, end-diastolic wall thickness.

Propagation error calculation

Assuming values for systolic pressure as 125±3.75 mmHg (16,665±500 Pa), LVIDD 52 ±1 mm (r_i of 26±0.5), EDWT 7.6±1 mm (r_o=33.6±0.5) and strain of 15.5±1% the result is 1.93±0.27 KJ/m^3 and the propagation error is 14%.

Source: https://www.eoas.ubc.ca/courses/eosc252/error-propagation-calculator-fj.htm)
